# Assessing the Effect of SNPs on Litter Traits in Pigs

**DOI:** 10.1155/2020/5243689

**Published:** 2020-07-14

**Authors:** Lyubov V. Getmantseva, Siroj Yu Bakoev, Varvara S. Shevtsova, Anatoly Yu Kolosov, Neckruz F. Bakoev, Maria A. Kolosova

**Affiliations:** ^1^Federal Science Center for Animal Husbandry named after Academy Member L.K. Ernst, Dubrovitsy 142132, Russia; ^2^Southern Federal University, Rostov-on-Don 344006, Russia; ^3^Don State Agrarian University, Persianovski 346493, Russia

## Abstract

The reproductive ability of sows is the principle of continuous and efficient production, based on such traits as the number of piglets, the total number of parities, and the period of economic use. Currently, SNPs associated with the TNB and NBA are presented in the PigQTLdb. The aim of this work was the assessment of the SNP effects on the litter traits in Large White (LW, *n* = 502) and Landrace (LN, *n* = 432) sow breeds in a farm in Russia. 9 SNPs (SNP_1: rs80956812; SNP_2: rs81471381; SNP_3: rs80891106; SNP_4: rs81399474; SNP_5: rs81421148; SNP_6: rs81242222; SNP_7: rs81319839; SNP_8: rs81312912; SNP_9: rs80962240) were selected for the study. Associative analysis was performed using the GLM procedure in R version 3.5.1. The analysis of reproductive traits was carried out according to the results of the first parity, the second and subsequent parities, and totals for lifetime of sows. The significant effect on litter traits in LW was determined for SNP rs80956812, SNP rs81471381, SNP rs81421148, and SNP rs81399474. The significant effect on litter traits in LN was determined for SNP rs81421148 and SNP rs81319839. *AKT3* gene was identified as perspective candidate gene, whose biological functions, as well as the results obtained in our work and in other studies, indicate its potential role in the reproductive process regulation in pigs. In general, the data obtained help to explain the genetic mechanisms of reproductive traits.

## 1. Introduction

Selection of specific individuals with desirable traits is fundamental for animal breeding. The scope and complexity of selection, as well as the number and size of populations, in traditional breeding programs require new tools based on recent advances in molecular biology and genetics. Therefore, the interest of scientists is focused on the study of the molecular genetic basis of the farm animal productive traits. Nowadays, genome-wide association study (GWAS) is the most powerful tool for studying polygenic traits genetic architecture [1, 2]. SNP genotyping panels are the most affordable solution for GWAS. These panels have been developed to include high (HD), medium (MD), or low (LD) density distribution of markers across the genome. Despite certain deficiencies [3], SNP panels have gained great popularity for the studies of quantitative trait genetic architecture in farm animals and pigs in particular [4–7]. One of the difficult tasks in pig breeding is improving the litter traits. The reproductive ability of sows is the principle of continuous and efficient production, based on such reproductive traits as the number of piglets, the total number of parities, and the period of economic use. The total number born (TNB) and the total number born alive (NBA) are the main characteristics of sows' litter traits [7–9]. These characteristics reflect the level of all physiological processes associated with fertilization, intrauterine development of fetuses, and birthing activity of sows and are easy to account for. In the last decades, the best linear unbiased prediction (BLUP) method has made a significant contribution to the improvement of the reproductive performance. However, the low heritability of reproductive traits (around 0.10) and their phenotypic manifestation limited by the sex of animals result in the need for developing new approaches to reveal the biological nature of reproductive abilities [10–12]. GWAS seems to be very useful for reproductive traits of genetic architecture studies. Currently, on the basis of genome map association results, 314 SNPs associated with TNB (225 SNPs) and NBA (89 SNPs) are presented in the PigQTLdb database [13]. The data obtained by GWAS allowed the identification of SNPs associated with litter traits in pigs. However, SNPs with signs of productivity may vary in different populations and also have a number of features due to the breed of pigs. For a better understanding of the SNP effects, it is necessary to conduct studies allowing the assessment of the repeatability of the obtained associations for different groups of pigs.

The aim of this work was to assess the effect of SNPs on litter traits in LW and LN pigs in a Russian breeding farm. To achieve this goal, it was necessary to select SNPs associated with TNB and NBA from the PigQTLdb and to develop techniques identifying these SNPs. Another goal was to identify prospective candidate genes to improve the reproductive qualities of pigs.

## 2. Methods

The research was carried out on the basis of the Center for Collective Use of Scientific Equipment “Bioresources and Bioengineering of Agricultural Animals” of the L.K. Ernst Federal Research Center for Animal Husbandry (https://www.vij.ru/infrastruktura/ckp). Samples from the Unique Scientific Installation (UNU) “Bank of Genetic Materials of Animals and Birds” (https://www.vij.ru/infrastruktura/46-infrastruktura/286-unikalnaya-nauchnaya-ustanovka-unu) were used for the work. For this collection, samples (tissue samples from the ear) were given by the owners of the breeding farms (according to their will). All methods were performed in accordance with the guidelines approved by the L.K. Ernst Federal Research Center for Animal Husbandry, Russia.

SNPs presented in the works of Sell-Kubiak et al. [14]; Bergfelder-Drüing et al. [15]; He et al. [16]; and Wang et al. [17] have been chosen for the study (Table 1). The names of these SNPs in accordance with the chips for genotyping on the basis of Ensembl Release 96 (April 2019) are presented in Add.  File 1. The primers were designed based on available genomic sequences presented in the National Center for Biotechnology Information (NCBI) database (plus/minus 300 bp from the certain SNP). Oligonucleotide primers and restriction endonucleases for polymerase chain reaction-restriction fragment length polymorphism (PCR-RFLP) identification of SNP were selected using the software Primer-BLAST (https://www.ncbi.nlm.nih.gov/tools/primer-blast/) and NEBcutter V2.0 (http://nc2.neb.com/NEBcutter2/index.php). In case of the restriction site absence, the pyrosequencing (PSQ) method was used. Selected primers, annealing temperature, and restriction endonuclease for PCR-RFLP are presented in Table 2.

The studies were carried out on LW (*n* = 502) and LN (*n* = 432) sow breeds in a farm in Russia. All the animals had the same conditions of housing and feeding. Genomic DNA was extracted from porcine tissue samples (ear aperture) using the DNA kit Extran-2 (Syntol, Russia) according to the manufacturer's instructions. The quantity, quality, and integrity of the DNA were assessed by Qubit 2.0 fluorimeter (Invitrogen/Life Technologies, USA) and a NanoDrop 8000 spectrophotometer (Thermo Fisher Scientific, USA).

The analysis of sows reproductive abilities was carried out according to the results of the first parity: total number born (TNB_1), total number born alive (NBA_1), litter weight of piglets born alive (BALWT_1); the results of the second and subsequent parities: total number born (TNB), total number born alive (NBA), litter weight of piglets born alive (BALWT); and totals for lifetime of sows: total number born (TNB_All), total number born alive (NBA_All), litter weight of piglets born alive (BALWT_All), and all parities (P_All). In accounting for P_All, the sows with at least three parities were selected.

## 3. Statistical Analysis

Associative analysis was performed using the GLM procedure in R version 3.5.1. The mixed model with the fixed effects was as follows: **Yij** **=** ***μ* ** **+ ** **Gi** **+** **Bj** **+** **eji**, where **Yij** is the ijth trait observation value; ***μ*** is the mean; **Gi** is the effect of the ith genotypes; **Bj** is the effect of jth breeding; and **eji** is the random residual that corresponds to the trait observation value. To determine if the SNP had a significant additive effect, dominance effect, or both, contrasts for additive and dominance effects were tested for the most significant SNP. Additive effects were declared when the contrast between the effects of the two homozygous genotypes was significantly (*P* ≤ 0.01 and *P* ≤ 0.05) or suggestively different (*P* ≤ 0.15). Dominance effects were declared when the contrast between the average effect of the two homozygous genotypes (AA and BB) and the effect of the heterozygous genotype was significantly (*P* ≤ 0.01 and *P* ≤ 0.05) or suggestively different (*P* ≤ 0.15). To adjust *P* values, the Bonferroni method was used.

Additive and dominant SNP effects were calculated using the following formulas [18]: *а* = (BB−AA)/2 and *d* = AB−(BB + AA)/2, where *a* is the additive effect; *d* is the dominant effect; and АА, АВ, and ВВ are the genotypes of SNP.

## 4. Results

The frequencies of alleles and genotypes of the studied SNP are presented in Table 3. LW pigs, in contrast to LN, were monomorphic on SNP_6. At the same time, polymorphism for SNP_1 in pigs LN was not detected. In addition, large differences in SNP_2, SNP_3, and SNP_7 between LW and LN should be noted. Further associative analysis was performed separately for the sows of LW and LN.

SNPs with the minor allele frequencies lower than 0.05 were excluded from the analysis. In LW, SNP_6 and SNP_8 were excluded; in LN, SNP_1 was excluded. The significant effect on litter traits in LW was determined for SNP_1 and SNP_2 on BALWT and for SNP_4 on BALWT_1 (Table 4). The suggestive effect on litter traits in LW was defined for SNP_3 on TNB_1, NBA_1, and P_All; for SNP_5 on TNB, NBA_1, BALWT_1, and BALWT; for SNP_7 on TNB_1; and for SNP_9 on BALWT.

The significant effects of SNP_5 on NBA and BALWT and of SNP_7 on NBA_1 and BALWT_1 in LN have been revealed (Table 5). The suggestive effect of SNP_2 on TNB_All and NBA_All, of SNP_5 on TNB_1 and TNB, and of SNP_7 on TNB_1 and BALWT in LN has been identified.

The results of additive and dominant effects of all studied SNPs are presented in Add.  File 2.

### 4.1. Additive and Dominant SNP Effects Specific to LW Sow

In general, the additive and dominant effects on litter traits in LW sow are determined for SNP_1, SNP_2, SNP_3, and SNP_5. The significant effect of the SNP_1 genotypes on BALWT is determined. The BALWT in the sows with the genotype SNP_1_BB was higher by 1.8 kg (*P* ≤ 0.05) and 1.1 kg (*P* ≤ 0.05) compared to the sows with the AA and AB genotypes, respectively. The negative dominant effect of SNP_2 on TNB and BALWT has been found. In the sows with the genotype SNP_2_AB, compared to homozygous sows, TNB and BALWT there were lesser by 0.7 (*P* ≤ 0.05) and 1.0 (*P* ≤ 0.05), respectively. In assessing the SNP_3 genotypes, additive effects on litter traits in first parity and the dominant effect on P_All were revealed. The sows with the genotype SNP_3_BB had 3.1 more TNB_1 and NBA_1 (*P* ≤ 0.05) compared to the sows of the genotype SNP_3_AA. For the entire productive period, sows with the genotype SNP_3_AB had 0.5 more P_All than sows with homozygous genotypes. According to the results of parities, the suggestive dominant effect on TNB_All, NBA_All, and BALWT_All is observed during the entire period of keeping. Analysis of the SNP_5 genotypes showed dominant effects on NBA_1 and BALWT_1, as well as additive effects on TNB, NBA, and BALWT. In the sows with the genotype SNP_5_AB, NBA_1 was smaller by 1.0 (*P* ≤ 0.05) and BALWT_1 was smaller by 1.6 (*P* ≤ 0.05) than these traits in the sows with homozygous genotype. Sows with the genotype SNP_5_BB had 1.1 more TNB (*P* ≤ 0.1), 0.9 more NBA (*P* ≤ 0.05), and 1.3 more BALWT (*P* ≤ 0.05).

### 4.2. Additive and Dominant SNP Effects Specific to LN Sow

We have determined the additive and dominant effects of SNP_2, SNP_5, and SNP_7 on litter traits of LN sows. The analysis of SNP_2 genotypes showed significant dominant effects on TNB_All and NBA_All and suggestive dominant effects on BALWT_All and P_All. Sows with genotype SNP_2_AB had 8.9 more TNB_All (*P* ≤ 0.05), 7.4 more NBA_All (*P* ≤ 0.05), and 0.5 more BALWT_All (*P* ≤ 0.1) and P_All (*P* ≤ 0.1) compared to homozygous sows. The additive effects of the genotypes SNP_5 were found for TNB, NBA, and BALWT. The sows with the genotype SNP_5_AA had 1.0 more TNB (*P* ≤ 0.05), 1.3 more NBA (*P* < 0.01), and 1.8 more BALWT (*P* < 0.01), compared to sows with the genotype SNP_5_BB. An investigation of the first parity showed significant effects of the SNP_7 genotypes on NBA_1 and BALWT_1. The sows with the genotype SNP_7_AB had 1.8 more NBA_1 than (*P* < 0.01), 1.1 more BALWT_1 (*P* < 0.01), and 1.4 more TNB_1 (*P* ≤ 0.1) compared to sows with the SNP_7_BB genotype. Significant effects of SNP_7 on subsequent parities were not observed statistically.

## 5. Discussion

Large White and Landrace breeds belong to the parent breeds used in three-breed breeding system of pigs at the first stage to obtain crossbreeding sows F1. Although the breeding work on the improvement of both LW and LN is mainly aimed at reproductive ability, each of these breeds has its own breed-specific characteristics, which are caused by differences in their genetic structure. In [15], Bergfelder-Drüing et al. revealed the genetic differentiation between LW and LN pigs. For GWAS, animals were divided into clusters by breeds, as well as interbreed clusters by the breeding farm. The results obtained on the basis of genotyping of the presented SNPs also showed the features of the frequencies and genotypes distribution associated with the pigs breed. To assess the effects of SNPs on litter traits, the animals under study were divided into two clusters according to the breed. Sell-Kubiak et al. [14] conducted GWAS for litter size in a LW pig population. According to their results, one of the significant SNPs associated with TNB was SNP rs80956812 (in our study, SNP_1) and SNP rs81471381 (in our study, SNP_2). In our studies, SNP_1 was monomorphic in LN. In LW, it has an additive effect on BALWT; effects on the other signs were not observed, possibly because of the low frequency of the SNP_1_AA genotype.

It is interesting to note that SNP_1 is localized in the intron of the small mothers against decapentaplegic 6 (*SMAD6)* gene (SSC1: 164,657,086–164,734,703; Sscrofa 11.1). The protein encoded by this gene belongs to the *SMAD* family of proteins, which have been identified as signaling mediators of the transforming growth factor beta superfamily (*TGF-beta*). They are involved in a number of biological processes, including cell growth, morphogenesis, development, and immune responses [19]. *SMAD1, SMAD2, SMAD3,* and *SMAD5* are ligand-specific: *SMAD1* and *SMAD5* transform signals from bone morphogenetic proteins (BMPs); *SMAD2* and *SMAD3* mediate TGF-beta signaling, and *SMAD4* acts as a common signaling component. *SMAD6* is completely different in structure from other *SMAD* proteins; it forms stable associations with type I receptors and works as an inhibitor [20].The great majority of *SMAD6* gene polymorphism studies are focused on identification of associations with human ovarian cancer [21, 22]. Based on these studies results, we can assume that genetic variations of the *SMAD6* gene can lead to changes in gene expression or regulation of the signaling function involved in the development of the reproductive process in pigs too.

Significant effects of SNP_2 on the analyzed traits were identified in LW and LN. It should be noted that these effects resulted from the heterozygous genotype (dominant effects). In LW, the effect of this SNP on BALWT only was determined, but in LN, effects were determined for all traits evaluated for the productive period (TNB_All, NBA_All, BALWT_All, P_ALL). SNP_2 is localized in the gene encoding hydroxymethylglutarate CoA-transferase succinate (*SUGCT,* SSC18: 53,639,593–54,283,251; Sscrofa 11.1). Sherman et al. [23] suggested that *SUGCT* (*C7orf10*) is a member of the coenzyme A family of class III transferases, based on a missense mutation (p.Arg336Trp) found in a homozygous state in several patients with type III glutaric aciduria. Further functional studies of catalytic activity and subcellular localization carried out by Marlaire et al. [24] confirmed that *SUGCT* (*C7orf10*) corresponds to succinyl-CoA: a mitochondrial enzyme glutarate CoA-transferase, involved in the metabolism of glutarate and possibly in the metabolism of longer dicarboxylic acids. Homologs of this enzyme are found in numerous bacterial operons, which also include the putative glutaryl CoA dehydrogenase, indicating that an enzyme with similar specificity exists in prokaryotes [24].

In studies of He et al. [16] on Chinese Erhualian pigs, 10 SNPs related to TNB and ovulation rate were presented. We have tested two of them, SNP rs80891106 (in our study, SNP_3) and SNP rs81399474 (in our study, SNP_4). Significant effects of SNP_3 on litter traits of first parity and on P_All were identified in LW. Possible functional features of SNP_3 are unknown yet, because of its localization in the noncoding region, and the nearby genes RF00001 (73,234,200–73,234,325; Sscrofa 11.1) and ENSSSCGG000032058 (73,861,910–73,911,853; Sscrofa 11.1) encode a ribosomal RNA and long noncoding RNA, respectively. However, data obtained by He et al. [16] and our results indicate the connection between SNP_3 and litter traits of pigs, whose mechanisms are still difficult to explain.

According to the LW pig research conducted by Wang et al. [17], we have chosen SNP rs81319839 (in our study, SNP_7), SNP rs81312912 (in our study, SNP_8), and SNP rs80962240 (in our study, SNP_9). In our work, the effects of SNP_7 on TNB_1 in LW and on NBA_1 and BALWT_1 in LN were established. This shows the significance of SNP_7 for the productivity of sows during the first farrowing period. In general, the first litter in the sows' life is associated with great stress for the body; productivity is realized on the background of the immature endocrine system of growing young animals [25]. In this regard, it is interesting to note that SNP_7 is localized in the SSC4 intergenic region, but one of the nearby genes is *MTBP* (SSC4: 18,535,841–18,609,163; Sscrofa 11.1). Brady et al. [26] showed the significant role of the *MTBP* gene (MDM2 binding protein) in the mechanisms ensuring the destruction of the p53 protein by the *MDM2* ubiquitinated ligase. The *p53* protein is a critical coordinator of a wide range of stress reactions in the body. The *p53* gene is expressed with constant activity, but the protein has a very short period of life, regulated by E3 ubiquitinated ligases. The most studied and probably significant of them is the ubiquitinated ligase *MDM2*. The *p53* regulation is provided by the negative feedback implemented by *MDM2* using *MTBP*. It is very important for the organism to regulate *p53* activity firmly and accurately to prevent inappropriate activation leading to the death of cell and possibly the death of organism [26]. This requires strict regulation of the correct balance between p53 and *MDM2*. Expression of *MDM2* is observed in a wide range of values and varies in different tissues, but the highest level is found in the testes and ovaries [27]. Interestingly, *MTBP* mRNA also has the highest level in these tissues [28]. In general, the role of *MTBP* in inhibiting the *cis*- and stimulating the *trans*-reactions of ubiquitin ligase E3 *MDM2* suggests that *MTBP* can also provide the functions which are necessary to regulate the reproductive activity of sows, especially during the first parity.

Among the studied SNPs, the most significant effect on variability of studied traits in the investigated population has been obtained for SNP_5 (SNP rs81421148). This result was described by Bergfelder-Drüing et al. [15] in their research on GWAS associated with the number of piglets born alive in LW sows. Our results have demonstrated significant association of SNP_5 with TNB, NBA, and BALWT in LW and LN. SNP_5 is localized in *AKT3* gene (SSC10: 16,441,465–16,741,745). This gene codes serine/threonine protein kinase. Three subtypes of protein kinases, AKT1, AKT2, and AKT3, have been identified in mammals; they play a key role in metabolism of glucose and angiogenesis as well as in PI3K/AKT signaling pathway, which regulates cell cycle and apoptosis. Despite the differences between the specific functions of *AKT* isoforms, all 3 subtypes are potential candidate genes associated with phenotype variation in humans and animals. The single-nucleotide polymorphism of *AKT3* (rs4590656) was found to be associated with three physiological parameters (hemoglobin, hematocrit, and red blood cell count) in people with chronic altitude sickness, indicating a strong association of this gene with angiogenesis [29]. A study by Gottlob et al. [30] showed that the level of *ATP* in the fibroblasts cell line in mice significantly depends on the *AKT* family. Bionaz and Loor [31] determined that the *AKT1* and *AKT3* levels of expression increase considerably during lactation periods in cattle. Wang et al. [32] revealed the effect of the *AKT3* gene polymorphism on the early maturity in rabbits. Chen et al. [33] demonstrated the gene polymorphism association with the components of muscle tissue in broilers. Liu et al. [34] found three insertions in *AKT3* gene associated with Xiang pig fattening traits.

The *AKT3* functional role is realized through the PI3K/AKT pathway. In mammals, an inhibition of the *PI3K/AKT* pathway blocks almost all insulin metabolic actions, including stimulation of glucose transport [35]. It is estimated that the *PI3K/AKT* pathway is involved in the regulation of folliculogenesis and oogenesis. The role of *AKT* in mammalian ovaries was evaluated by gene knockout. Female mice with *AKT1* deficiency have demonstrated that the fertility decreases, the estrus delays for about 5 days, the age of the first litter increases, and the average litter size decreases. Primary follicles oocytes in females with *AKT1* deficiency are larger than those in wild-type animals, and sometimes the follicles contain several oocytes. The effects of *AKT2* and *AKT3* on fertility are still unclear. The AKT controls the activity of some transcription factors of the forkhead box protein O1 or *FOXO* family (*FOXO1, FOXO3,* and *FOXO4*). The transcription factors *FOXO1*, *FOXO3*, and *FOXO4* are also involved in the control of folliculogenesis [36]. On the basis of all the above, it can be assumed that *AKT3* is a perspective gene candidate for reproductive traits of pigs.

## 6. Conclusion

The results obtained showed the pleiotropic effect of the selected SNPs on the traits related to the litter size, the litter weight, and the amount of parities. Here, we demonstrated the local genotyping systems of some SNPs from PigQTLdb, which can be used in further studies on the genetic architecture and its connection with the pig productivity traits. The analysis of the SNPs alleles and genotypes frequency distribution revealed features associated with the pigs' breed, which must be considered when evaluating the effects of SNPs. The results showed significant effects of SNPs on litter traits in LW and LN. *AKT3* was identified as a promising candidate gene, whose biological functions, as well as the results obtained in our work and in other studies, indicate its potential role in the reproductive process regulation in pigs. In general, the data obtained help to explain the genetic mechanisms of reproductive traits.

## Figures and Tables

**Table 1:** Selected SNPs for the study.

Number of SNPs (in text)	SNP	SSC	Location		Genes	Reference (breed)
			Sscrofa 11.1	Sscrofa 10.2		
SNP_1	rs80956812	1	164674664	182418248	*SMAD6*	[14]
SNP_2	rs81471381	18	53672799	58861041	*SUGCT*	[14]
SNP_3	rs80891106	7	73467314	78538720	—	[16]
SNP_4	rs81399474	8	32370687	33985796	—	[16]
SNP_5	rs81421148	10	16506621	18203672	*AKT3*	[15]
SNP_6	rs81242222	11	67129570	74240078	—	[15]
SNP_7	rs81319839	4	18194352	19239772	—	[17]
SNP_8	rs81312912	4	18196598	19237526		[17]
SNP_9	rs80962240	13	52784022	58478836	*FOXP1*	[17]

**Table 2:** Oligonucleotide primers and restriction endonucleases to identify the SNPs under study.

Number of SNPs	SNP	Oligonucleotide primers	Annealing temperature	Length of amplicon (bp)	Restriction endonuclease/PSQ	Length of restriction fragments (bp)
SNP_1	rs80956812	F5′-GGCGCTAAGTGAGCTCTTG-3′ R5′-CCCTCACTGATGCAACTCTAAA-3′		296	Bme18 I	202, 94

SNP_2	rs81471381	F5′-CCACGCTCTCTACAAGCCAA-3′ R5′-CCCCATTCACGGTCTTGGAA-3′		540	SfaN I	368, 172

SNP_3	rs80891106	F5′-TGACAAGCTTCAGACAGTTCCT-3′ R5′-TGCACTGAACCTTCACACACA-3′		332	PSQ	

SNP_4	rs81399474	F5′-AGAACGAGGCTTCTTCCTGTT-3′ R5′-ACAGTCTAAAGCCTGATTTCCCT-3′		297	PSQ	

SNP_5	rs81421148	F5′-TTCTGTACTTCTCCATCACAAGAA-3′ R5′-CGAAGACTTGTTTACGCATCATAG-3′		383	Mnl I	276, 107

SNP_6	rs81242222	F5′-TGCAGAGATTCCAGCAAGCC-3′ R5′-CATCTGGTTGGTTTGGTCGTG-3′		290	PSQ	

SNP_7	rs81319839	F5′-GAAGCACCCAATGGGACTCT-3′ R5′-ATGAGGTTGTCTTGGCACCAT-3′		356	BstMB I	276, 80

SNP_8	rs81312912	F5′-ACAGGACAGTATGAAAAATCTGTTG-3′ R5′-GCTTCCCCCAGAAAGGACTG-3′		302	BstAU I	241, 61

SNP_9	rs80962240	F5′-ATGGAGGAACCGGCTATGTG-3′ R5′-GCAGTCCTGCCCATGAGTAT-3′		461	Vsp I	350, 111

**Table 3:** Frequency of alleles and genotypes.

SNP	Genotype						Allele	
	AA		AB		BB		A	B
	*n*	%	*n*	%	*n*	%		
LW (*n* = 502)								
SNP_1	25	4.98	216	43.03	261	51.99	0.26	0.74
SNP_2	80	15.94	201	40.04	221	44.02	0.36	0.64
SNP_3	10	1.99	141	28.09	351	69.92	0.16	0.84
SNP_4	15	2.99	196	39.04	291	57.97	0.23	0.77
SNP_5	105	20.92	263	52.39	134	26.69	0.47	0.53
SNP_6	0	0.00	0	0.00	502	100.00	0.00	1.00
SNP_7	402	80.08	95	18.92	5	1.00	0.90	0.10
SNP_8	462	92.03	35	6.97	5	1.00	0.96	0.04
SNP_9	151	30.08	276	54.98	75	14.94	0.58	0.42

LN (*n* = 432)								
	AA		AB			BB	A	B

SNP_1	0	0.00	0	0.00	432	100.00	0.00	1.00
SNP_2	207	47.92	203	46.99	22	5.09	0.71	0.29
SNP_3	211	48.84	178	41.20	43	9.95	0.69	0.31
SNP_4	34	7.87	199	46.06	199	46.06	0.31	0.69
SNP _5	99	22.92	229	53.01	104	24.07	0.49	0.51
SNP_6	9	2.08	147	34.03	276	63.89	0.19	0.81
SNP_7	0	0.00	60	13.89	372	86.11	0.07	0.93
SNP_8	216	50.00	186	43.06	30	6.94	0.72	0.28
SNP_9	268	62.04	112	25.93	52	12.04	0.75	0.25

**Table 4:** The SNP effects on the litter traits in LW.

Traits	SNP_1	SNP_2	SNP_3	SNP_4	SNP_5	SNP_7	SNP_9
TNB_1	0.665	0.616	0.122	0.239	0.282	0.062	0.204
TNB	0.976	0.189	0.710	0.383	0.100	0.686	0.730
TNB_All	0.902	0.762	0.251	0.911	0.725	0.561	0.755
NBA_1	0.662	0.636	0.108	0.181	0.082	0.312	0.214
NBA	0.765	0.202	0.745	0.264	0.149	0.837	0.733
NBA_All	0.993	0.936	0.306	0.840	0.820	0.501	0.749
BALWT _1	0.620	0.304	0.191	**0.033** ^*∗*^	0.060	0.562	0.551
BALWT	**0.034** ^*∗*^	**0.046** ^*∗*^	0.830	0.434	0.100	0.976	0.074
BALWT _All	0.685	0.695	0.190	0.825	0.743	0.486	0.992
P_All	0.933	0.968	0.129	0.683	0.738	0.425	0.537

^*∗*^ *P* < 0.05. SNP_1: rs80956812; SNP_2: rs81471381; SNP_3: rs80891106; SNP_4: rs81399474; SNP_5: rs81421148; SNP_7: rs81319839; SNP_9: rs80962240. TNB_1: total number born of the first parity; TNB: total number born of the second and subsequent parities; TNB_All: total number born of lifetime sow; NBA_1: total number born alive of the first parity; NBA: total number born alive of the second and subsequent parities; NBA_All: total number born alive of lifetime sow; BALWT_1: litter weight of piglets born alive of the first parity; BALWT: litter weight of piglets born alive of the second and subsequent parities; BALWT_All: litter weight of piglets born alive of lifetime sow; P_All: all parities of lifetime sow.

**Table 5:** The SNP effects on the litter traits in LN.

	SNP_2	SNP_3	SNP_4	SNP_5	SNP_6	SNP_7	SNP_8	SNP_9
TNB_1	0.193	0.446	0.661	0.137	0.716	0.067	0.315	0.655
TNB	0.325	0.299	0.533	0.079	0.985	0.638	0.367	0.296
TNB_All	0.087	0.846	0.727	0.415	0.827	0.242	0.578	0.303
NBA_1	0.370	0.513	0.487	0.196	0.833	**0.014** ^*∗*^	0.718	0.339
NBA	0.375	0.300	0.337	**0.022** ^*∗*^	0.836	0.152	0.642	0.480
NBA_All	0.109	0.809	0.754	0.404	0.755	0.151	0.385	0.289
BALWT _1	0.369	0.700	0.476	0.174	0.684	**0.005** ^*∗∗*^	0.872	0.227
BALWT	0.544	0.414	0.311	**0.029** ^*∗*^	0.807	0.144	0.727	0.634
BALWT _All	0.208	0.856	0.889	0.444	0.755	0.208	0.395	0.290
P_All	0.296	0.990	0.595	0.633	0.757	0.197	0.247	0.354

^*∗*^ *P* < 0.05, ^*∗∗*^*P* < 0.01. SNP_2: rs81471381; SNP_3: rs80891106; SNP_4: rs81399474; SNP_5: rs81421148; SNP_6: rs81242222; SNP_7: rs81319839; SNP_8: rs81312912; SNP_9: rs80962240. TNB_1: total number born of the first parity; TNB: total number born of the second and subsequent parities; TNB_All: total number born of lifetime sow; NBA_1: total number born alive of the first parity; NBA: total number born alive of the second and subsequent parities; NBA_All: total number born alive of lifetime sow; BALWT_1: litter weight of piglets born alive of the first parity; BALWT: litter weight of piglets born alive of the second and subsequent parities; BALWT_All: litter weight of piglets born alive of lifetime sow; P_All: all parities of lifetime sow.

## Data Availability

The datasets produced and/or analyzed during the current study are available from the corresponding author on reasonable request.

## Abbreviations

QTL: Quantitative trait loci
GWAS: Genome-wide association study
SNP: Single-nucleotide polymorphism
TNB: Total number born
NBA: Total number born alive
PigQTLdb: Pig quantitative trait loci database
PCA: Principal component analysis
LW: Large White
LN: Landrace
AKT3: AKT serine/threonine kinase 3 gene
BLUP: Best linear unbiased prediction
NCBI: National Center for Biotechnology Information
PCR-RFLP: Polymerase chain reaction-restriction fragment length polymorphism
PSQ: Pyrosequencing method
BALWT: Litter weight of piglets born alive
TNB_1: Total number born of the first parity
NBA_1: Total number born alive of the first parity
BALWT_1: Litter weight of piglets born alive of the first parity
TNB_All: Total number born of the lifetime sow
NBA_All: Total number born alive of the lifetime sow
BALWT_All: Litter weight of piglets born alive of the lifetime sow
P_All: All parities of the lifetime sow
SMAD: Small mothers against decapentaplegic
SSC: *Sus scrofa* chromosome.

## Additional Points

*Highlights*. The effect on litter traits in LW was determined for SNPs rs80956812, rs81471381, rs81421148, and rs81399474. The effect on litter traits in LN was determined for SNPs rs81421148 and rs81319839. The AKT3 gene can be considered as a potential candidate gene. Local genotyping systems of some SNPs from PigQTLdb can be used in further studies.

## Supplementary Materials

Supplementary Materials Add. File 1: the names of the SNPs in accordance with the chips for genotyping on the basis of Ensembl Release 96 (April 2019). Add. File 2: Table F2_1—the results of additive and dominant effects of SNPs in LW; Table F2_2—the results of additive and dominant effects of SNPs in LN.
