# Supplementary material for: Assessing the Effect of SNPs on Litter Traits in Pigs
Source: Scientifica (Cairo). 2020 Jul 14;2020:5243689. doi: 10.1155/2020/5243689 (PMC7414332; doi:10.1155/2020/5243689)
Supplement: Supplementary Materials — Add. File 1: the names of the SNPs in accordance with the chips for genotyping on the basis of Ensembl Release 96 (April 2019). Add. File 2: Table F2_1—the results of additive and dominant effects of SNPs in LW; Table F2_2—the results of additive and dominant effects of SNPs in LN. [file 5243689.f1.doc]

Add. File 1. The name of these SNPs in accordance with the chips for genotyping on the basis of EnsemblRelease 96 (April 2019)

| Number of SNP (in text) | SNP | HGVS names | Axiom Genotyping Array | GGP Porcine HD | GGP Porcine LD | Pig SNP Consortium  (Illumina_PorcineSNP60) |
| --- | --- | --- | --- | --- | --- | --- |
| SNP_1 | rs80956812 | NC_010443.5:g.164674664G>A | WU_10_2_1_182418248 |  |  | ALGA0006771 |
| SNP_2 | rs81471381 | NC_010460.4:g.53672799A>G | WU_10_2_18_58861041 | ALGA0098906 |  | ALGA0098906 |
| SNP_3 | rs80891106 | NC_010449.5:g.73467314G>A | WU_10_2_7_78538720 | ALGA0042559 | ALGA0042559 | ALGA0042559 |
| SNP_4 | rs81399474 | NC_010450.4:g.32370687T>C | WU_10_2_8_33985796 |  |  | ALGA0047440 |
| SNP_5 | rs81421148 | NC_010452.4:g.16506621A>C | WU_10_2_10_18203672 |  | ASGA0046811 | ASGA0046811 |
| SNP_6 | rs81242222 | NC_010453.5:g.67129570G>A | WU_10_2_11_74240078 | MARC0006510 |  | MARC0006510 |
| SNP_7 | rs81319839 | NC_010446.5:g.18194352G>A | WU_10_2_4_19239772 | ASGA0099361 |  | ASGA0099361 |
| SNP_8 | rs81312912 | NC_010446.5:g.18196598G>A | WU_10_2_4_19237526 | ASGA0093900 |  | ASGA0093900 |
| SNP_9 | rs80962240 | NC_010455.5:g.52784022T>C | WU_10_2_13_58478836 | ALGA0070192 | ALGA0070192 | ALGA0070192 |

add. File 2.

Table F2_1. The results of additive and dominant effects of SNPs in LW

| Traits | | | Genotypes | | | | | | | Effect | | | | | |
| --- | --- | --- | --- | --- | --- | --- | --- | --- | --- | --- | --- | --- | --- | --- | --- |
|  |  |  | АА | | | АВ | | | ВВ | АА-ВВ | | a | | d | |
| SNP_1 (rs80956812) | | | | | | | | | | | | | | | |
| TNB_1 | 11.85±1.09 | | | 11.39 ±1.15 | | | 11.05± 1.14 | | | 0.80 | | 0.40 | | | 0.27 |
| TNB | 14.08±0.79 | | | 13.92±0.84 | | | 13.97±0.83 | | | 0.11 | | 0.06 | | | -0.06 |
| TNB_All | 81.86±9.24 | | | 79.77±9.84 | | | 78.29±9.72 | | | 3.57 | | 1.79 | | | 1.14 |
| NBA_1 | 11.00±1.13 | | | 9.81±1.91 | | | 9.82±1.18 | | | 1.18 | | 0.59 | | | -0.09 |
| NBA | 12.39±0.75 | | | 12.84±0.80 | | | 12.95±0.79 | | | -0.56 | | -0.28 | | | -0.04 |
| NBA_All | 72.71±8.82 | | | 71.94±9.40 | | | 71.68±9.28 | | | 1.03 | | 0.52 | | | 0.16 |
| BALWT_1 | 12.85±1.53 | | | 12.86±1.62 | | | 12.29±1.61 | | | 0.56 | | 0.28 | | | 0.53 |
| BALWT | 14.32±0.97 | | | 15.05±1.04 | | | 16.12±1.02 | | | **-1.80*** | | **-0.90*** | | | **-0.89*** |
| BALWT_All | 84.29±10.19 | | | 84.89±10.85 | | | 89.03±10.72 | | | -4.74 | | -2.37 | | | -3.67 |
| P_All | 6.00±0.54 | | | 5.89±0.57 | | | 5.82±0.57 | | | 0.18 | | 0.09 | | | +0.05 |
| SNP_2 (rs81471381) | | | | | | | | | | | | | | | |
| TNB_1 | 10.75±0.59 | | | 11.45±0.69 | | | 11.22±0.69 | | | -0.47 | | -0.24 | | | +0.36 |
| TNB | 14.12±0.44 | | | 13.68±0.42 | | | 14.28±0.53 | | | -0.16 | | -0.08 | | | **-0.66*** |
| TNB_All | 78.15±5.46 | | | 77.69±6.37 | | | 81.04±6.46 | | | -2.89 | | -1.45 | | | -2.52 |
| NBA_1 | 9.50±0.61 | | | 10.17±0.72 | | | 9.73±0.71 | | | -0.23 | | -0.12 | | | +0.50 |
| NBA | 13.29±0.42 | | | 12.53±0.50 | | | 13.08±0.50 | | | 0.21 | | 0.11 | | | ***-0.61*** |
| NBA_All | 72.70±5.22 | | | 71.00±6.09 | | | 72.44±6.17 | | | 0.26 | | 0.13 | | | -1.51 |
| BALWT_1 | 12.79±0.83 | | | 12.89±0.98 | | | 12.18±0.97 | | | 0.61 | | 0.31 | | | +0.55 |
| BALWT | 16.61±0.55 | | | 15.01±0.66 | | | 15.73±0.66 | | | 0.88 | | 0.44 | | | **-0.97*** |
| BALWT_All | 91.20±6.03 | | | 85.24±7.04 | | | 87.38±7.13 | | | 3.82 | | 1.91 | | | -3.24 |
| P_All | 5.85±0.32 | | | 5.89±0.37 | | | 5.82±0.38 | | | 0.03 | | 0.02 | | | +0.06 |
| SNP_3 (rs80891106) | | | | | | | | | | | | | | | |
| TNB_1 | 8.33±1.64 | | | 10.95±1.71 | | | 11.44±1.67 | | | | **-3.11*** | | **-1.56*** | | -0.40 |
| TNB | 12.89±1.35 | | | 13.93±1.39 | | | 14.00±1.36 | | | | -1.11 | | -0.56 | | -0.04 |
| TNB_All | 72.50±17.10 | | | 84.41±17.54 | | | 76.79±17.31 | | | | -4.29 | | -2.15 | | ***7.71*** |
| NBA_1 | 7.00±1.71 | | | 9.51±1.73 | | | 10.09±1.46 | | | | **-3.09*** | | **-1.55*** | | -0.49 |
| NBA | 12.04±1.28 | | | 12.79±1.32 | | | 12.94±1.30 | | | | -0.90 | | -0.45 | | -0.11 |
| NBA_All | 62.00±16.35 | | | 76.36±16.76 | | | 69.99±16.54 | | | | -7.99 | | -4.00 | | ***6.56*** |
| BALWT_1 | 9.33±2.32 | | | 11.89±2.41 | | | 12.92±2.35 | | | | -3.59 | | -1.80 | | -0.93 |
| BALWT | 14.58±1.70 | | | 15.55±1.75 | | | 15.61±1.03 | | | | -1.03 | | -0.52 | | -0.02 |
| BALWT_All | 72.00±18.86 | | | 93.15±19.34 | | | 84.57±19.08 | | | | -12.57 | | -6.29 | | ***8.88*** |
| P_All | 6.00±0.99 | | | 6.23±1.01 | | | 5.68±1.01 | | | | 0.32 | | 0.16 | | **0.54*** |
| SNP_4 (rs81399474) | | | | | | | | | | | | | | | |
| TNB_1 | 13.20±1.28 | | | 11.00±1.34 | | | 11.28±1.32 | | | ***1.92*** | | | ***0.96*** | | -0.39 |
| TNB | 15.02±1.11 | | | 13.71±1.15 | | | 14.08±1.14 | | | 0.94 | | | 0.47 | | 0.41 |
| TNB_All | 76.50±12.22 | | | 78.20±12.70 | | | 79.89±12.56 | | | -3.39 | | | -1.70 | | -1.51 |
| NBA_1 | 12.00±1.32 | | | 9.57±1.38 | | | 9.94±1.36 | | | ***2.06*** | | | ***1.03*** | | -0.48 |
| NBA | 14.07±1.05 | | | 12.60±1.09 | | | 13.02±1.08 | | | ***1.05*** | | | ***0.53*** | | -0.46 |
| NBA_All | 69.75±11.66 | | | 70.50±12.11 | | | 72.92±11.98 | | | -3.17 | | | -1.59 | | -2.25 |
| BALWT_1 | 16.80±1.78 | | | 12.00±1.86 | | | 12.70±1.83 | | | **4.10*** | | | **2.05*** | | -0.95 |
| BALWT | 16.60±1.41 | | | 15.25±1.46 | | | 15.76±1.44 | | | 0.84 | | | 0.42 | | -0.54 |
| BALWT_All | 84.00±13.50 | | | 85.46±14.03 | | | 88.34±13.88 | | | -4.34 | | | -2.17 | | -2.65 |
| P_All | 5.25±0.71 | | | 5.86±0.74 | | | 5.89±0.73 | | | -0.64 | | | -0.32 | | -0.01 |
| SNP_5 (rs81421148) | | | | | | | | | | | | | | | |
| TNB_1 | 11.63±0.52 | | | 10.90±0.62 | | | 11.61±0.70 | | | 0.02 | | | 0.01 | | ***-0.73*** |
| TNB | 13.41±0.41 | | | 13.86±0.48 | | | 14.53±0.54 | | | **-1.12*** | | | **-0.56*** | | -0.19 |
| TNB_All | 78.04±5.09 | | | 78.03±5.89 | | | 81.97±6.59 | | | -3.93 | | | -1.97 | | -2.36 |
| NBA_1 | 10.66±0.53 | | | 9.39±0.63 | | | 10.20±0.71 | | | 0.46 | | | 0.23 | | **-1.01*** |
| NBA | 12.52±0.39 | | | 12.74±0.45 | | | 13.42±0.52 | | | **-0.90*** | | | **-0.45*** | | -0.29 |
| NBA_All | 70.96±4.86 | | | 71.07±5.62 | | | 74.00±6.30 | | | -3.04 | | | -1.52 | | -1.70 |
| BALWT_1 | 13.53±0.72 | | | 11.81±0.86 | | | 13.33±0.97 | | | 0.20 | | | 0.10 | | **-1.62*** |
| BALWT | 15.11±0.52 | | | 15.33±0.61 | | | 16.37±0.68 | | | **-1.26*** | | | **0.63*** | | -0.51 |
| BALWT_All | 87.13±5.63 | | | 85.57±6.51 | | | 89.94±7.28 | | | -2.81 | | | -1.41 | | -3.23 |
| P_All | 6.04±0.30 | | | 5.78±0.34 | | | 5.88±0.38 | | | -0.16 | | | -0.08 | | -0.17 |
| SNP_7 (rs81319839) | | | | | | | | | | | | | | | |
| TNB_1 | | 11.51±0.26 | | | 10.11±0.61 | | | 10.50±2.02 | | 1.01 | | | 0.55 | | **-1.38*** |
| TNB | | 14.02±0.21 | | | 13.66±0.47 | | | 14.49±1.44 | | -0.47 | | | -0.24 | | -0.37 |
| TNB_All | | 78.79±2.45 | | | 78.88±5.54 | | | 97.50±17.39 | | -18.71 | | | -9.36 | | -0.28 |
| NBA_1 | | 10.03±0.28 | | | 9.11±0.63 | | | 10.49±2.12 | | -0.46 | | | -0.23 | | ***-0.93*** |
| NBA | | 12.91±0.19 | | | 12.72±0.44 | | | 13.41±1.37 | | -0.50 | | | -0.25 | | -0.27 |
| NBA_All | | 71.45±2.33 | | | 71.87±5.28 | | | 90.99±16.58 | | -19.54 | | | -9.77 | | +0.03 |
| BALWT_1 | | 12.73±0.37 | | | 11.96±0.86 | | | 11.00±2.89 | | 1.73 | | | 0.87 | | -0.74 |
| BALWT | | 15.56±0.26 | | | 15.65±0.59 | | | 15.83±1.83 | | -0.27 | | | -0.14 | | +0.09 |
| BALWT_All | | 85.99±2.70 | | | 89.79±6.12 | | | 106.50±19.20 | | -20.51 | | | 10.26 | | -3.40 |
| P_All | | 5.80±0.14 | | | 6.00±0.32 | | | 7.00±1.01 | | -1.20 | | | 0.60 | | -0.18 |
| SNP_9 (rs80962240) | | | | | | | | | | | | | | | |
| TNB_1 | | 10.65±0.43 | | | 11.58±0.53 | | | 11.08±0.75 | | -0.43 | | | -0.22 | | ***+0.75*** |
| TNB | | 14.14±0.34 | | | 13.83±0.42 | | | 14.09±0.59 | | 0.05 | | | 0.03 | | -0.29 |
| TNB_All | | 76.70±4.01 | | | 80.43±4.97 | | | 78.95±6.89 | | -2.25 | | | -1.13 | | +2.97 |
| NBA_1 | | 9.41±0.44 | | | 10.24±0.55 | | | 9.39±0.78 | | 0.02 | | | 0.01 | | ***+0.84*** |
| NBA | | 13.07±0.32 | | | 12.77±0.40 | | | 12.97±0.56 | | 0.10 | | | 0.05 | | -0.27 |
| NBA_All | | 69.41±3.83 | | | 72.95±4.75 | | | 72.63±6.57 | | -3.23 | | | -1.62 | | +2.44 |
| BALWT_1 | | 12.29±0.61 | | | 12.88±0.76 | | | 11.90±1.08 | | 0.39 | | | 0.20 | | 0.71 |
| BALWT | | 16.37±0.43 | | | 15.17±0.52 | | | 15.63±0.74 | | 0.74 | | | 0.37 | | **-0.95*** |
| BALWT_All | | 87.38±4.45 | | | 87.04±5.51 | | | 86.41±7.63 | | 0.96 | | | 0.48 | | -0.01 |
| P_All | | 5.65±0.23 | | | 5.97±0.29 | | | 5.84±0.40 | | -0.19 | | | -0.10 | | +0.25 |

** - P ≤ 0.01; * - P ≤ 0.05; bold italics P ≤ 0.15

Table F2_2. The results of additive and dominant effects of SNPs in LN

| Traits | | Genotypes | | | | | Effect | | | |
| --- | --- | --- | --- | --- | --- | --- | --- | --- | --- | --- |
|  |  | АА | | АВ | ВВ | | АА-ВВ | a | d | |
| SNP_2 (rs81471381) | | | | | | | | | | |
| TNB_1 | 12.96±0.37 | | 13.33±0.53 | | | 11.20±1.20 | 1.76 | 0.88 | | 0.54 |
| TNB | 13.87±0.45 | | 14.34±0.38 | | | 14.71±0.81 | -0.84 | -0.42 | | 0.35 |
| TNB_All | 63.53±3.04 | | 72.93±4.19 | | | 68.60±8.70 | -5.07 | -2.54 | | **8.78*** |
| NBA_1 | 11.34±0.39 | | 11.26±0.55 | | | 9.60±1.26 | 1.74 | 0.87 | | 0.08 |
| NBA | 12.56±0.37 | | 12.94±0.34 | | | 13.31±0.73 | -0.75 | -0.38 | | 0.31 |
| NBA_All | 57.19±2.70 | | 65.15±3.72 | | | 61.60±7.73 | -4.41 | -2.21 | | **7.42*** |
| BALWT_1 | 16.57±0.57 | | 16.21±0.82 | | | 14.00±1.85 | ***2.57*** | 1.29 | | -0.11 |
| BALWT | 18.54±0.58 | | 18.80±0.51 | | | 19.67±1.09 | -1.13 | -0.57 | | 0.40 |
| BALWT_All | 84.72±4.03 | | 94.65±5.56 | | | 91.40±11.54 | -6.68 | -3.34 | | ***9.11*** |
| P_All | 4.81±0.18 | | 5.21±0.33 | | | 4.82±0.42 | -0.01 | -0.01 | | ***0.46*** |
| SNP_3 (rs80891106) | | | | | | | | | | |
| TNB_1 | 13.23±0.38 | | 13.04±0.55 | | | 12.11±0.89 | 1.12 | 0.56 | | 0.06 |
| TNB | 14.28±0.44 | | 14.18±0.39 | | | 13.26±0.68 | ***1.02*** | ***0.51*** | | 0.24 |
| TNB_All | 69.50±2.97 | | 67.91±4.38 | | | 65.43±7.69 | 4.07 | 2.04 | | -0.98 |
| NBA_1 | 11.33±0.38 | | 11.30±0.57 | | | 10.30±0.93 | 1.03 | 0.52 | | 0.14 |
| NBA | 12.88±0.36 | | 12.87±0.35 | | | 11.98±0.61 | ***0.90*** | ***0.45*** | | 0.27 |
| NBA_All | 62.23±2.63 | | 61.15±3.88 | | | 57.86±6.81 | 4.37 | 2.19 | | -0.43 |
| BALWT_1 | 16.44±0.57 | | 16.33±0.84 | | | 15.30±1.98 | 1.14 | 0.57 | | 0.08 |
| BALWT | 18.93±0.57 | | 18.74±0.53 | | | 17.74±0.91 | ***1.19*** | ***0.60*** | | 0.20 |
| BALWT_All | 91.33±3.89 | | 89.33±5.75 | | | 86.15±10.09 | 5.18 | 2.59 | | -1.23 |
| P_All | 4.93±0.20 | | 4.95±0.30 | | | 5.01±0.53 | -0.08 | -0.04 | | 0.01 |
| SNP_4 (rs81399474) | | | | | | | | | | |
| TNB_1 | 12.93±0.92 | | 12.82±0.99 | | | 13.28±1.00 | -0.35 | -0.18 | | -0.45 |
| TNB | 13.70±0.78 | | 14.02±0.33 | | | 14.37±0.74 | -0.67 | 0.34 | | -0.17 |
| TNB_All | 67.33±7.64 | | 70.80±8.21 | | | 67.40±8.26 | 0.53 | 0.27 | | 3.32 |
| NBA_1 | 11.50±0.95 | | 10.87±1.03 | | | 11.51±1.03 | -0.01 | -0.01 | | -0.64 |
| NBA | 12.15±0.68 | | 12.69±0.66 | | | 13.03±0.67 | -0.88 | -0.44 | | -0.06 |
| NBA_All | 59.33±6.78 | | 62.82±7.28 | | | 60.19±7.32 | -0.86 | -0.43 | | 2.75 |
| BALWT_1 | 16.63±1.40 | | 15.76±1.51 | | | 16.74±1.51 | -0.11 | -0.06 | | -0.96 |
| BALWT | 18.34±1.04 | | 18.42±0.99 | | | 19.19±0.99 | -0.85 | -0.43 | | -0.55 |
| BALWT_All | 89.17±10.06 | | 91.41±10.81 | | | 88.70±10.87 | 0.47 | 0.24 | | 2.65 |
| P_All | 5.00±0.52 | | 5.08±0.56 | | | 4.78±0.56 | 0.22 | 0.11 | | 0.27 |
| SNP_5 (rs81421148) | | | | | | | | | | |
| TNB_1 | 12.26±0.53 | | 13.49±0.64 | | | 12.86±0.74 | -0.60 | -0.30 | | ***0.94*** |
| TNB | 14.84±0.51 | | 13.96±0.45 | | | 13.80±0.52 | **1.04*** | 0.52 | | -0.41 |
| TNB_All | 72.95±4.16 | | 66.19±5.07 | | | 68.70±5.88 | 4.25 | 2.13 | | -4.63 |
| NBA_1 | 10.35±0.55 | | 11.49±0.67 | | | 11.46±0.77 | -1.11 | -0.56 | | 0.58 |
| NBA | 13.50±0.42 | | 12.20±0.40 | | | 12.25±0.46 | **1.25**** | 0.63 | | -0.15 |
| NBA_All | 65.60±3.69 | | 59.54±4.50 | | | 61.00±5.21 | 4.60 | 2.30 | | -3.76 |
| BALWT_1 | 14.96±0.81 | | 16.63±0.98 | | | 16.80±1.14 | ***-1.84*** | ***-0.92*** | | 0.73 |
| BALWT | 19.73±0.67 | | 18.64±0.60 | | | 17.90±0.69 | **1.83**** | **0.92**** | | -0.14 |
| BALWT_All | 95.85±5.46 | | 87.32±6.66 | | | 89.80±7.72 | 6.05 | 3.01 | | -5.51 |
| P_All | 5.10±0.29 | | 4.80±0.35 | | | 5.05±0.40 | 0.05 | 0.03 | | -0.27 |
| SNP_6 (rs81242222) | | | | | | | | | | |
| TNB_1 | 14.86±2.58 | | 12.84±2.66 | | | 13.03±2.59 | 1.83 | 0.92 | | -0.24 |
| TNB | 14.48±1.89 | | 14.14±1.92 | | | 14.15±1.86 | 0.33 | 0.17 | | 0.06 |
| TNB_All | 57.00±18.75 | | 68.44±19.77 | | | 68.65±19.88 | -11.65 | -5.83 | | -0.04 |
| NBA_1 | 12.00±2.69 | | 11.58±2.80 | | | 11.15±2.71 | 0.85 | 0.43 | | 0.42 |
| NBA | 13.27±1.71 | | 13.09±1.74 | | | 12.75±1.69 | 0.52 | 0.26 | | 0.33 |
| NBA_All | 51.00±16.61 | | 63.67±17.51 | | | 61.25±16.73 | -10.25 | -5.13 | | 2.56 |
| BALWT_1 | 17.00±3.96 | | 17.17±0.17 | | | 16.14±3.98 | 0.86 | 0.43 | | 1.02 |
| BALWT | 19.68±2.56 | | 19.18±2.60 | | | 18.68±2.53 | 1.00 | 0.50 | | 0.20 |
| BALWT_All | 75.00±24.59 | | 93.56±25.92 | | | 89.80±24.76 | -14.80 | -7.40 | | 3.96 |
| P_All | 4.00±1.28 | | 5.00±1.35 | | | 4.94±1.29 | -0.94 | -0.47 | | 0.07 |
| SNP_7 (rs81319839) | | | | | | | | | | |
| TNB_1 | - | | 14.20±0.68 | | | 12.80±0.74 | ***1.40*** |  | |  |
| TNB | - | | 13.93±0.63 | | | 14.19±0.55 | -0.26 |  | |  |
| TNB_All | - | | 62.67±5.35 | | | 69.50±5.79 | -6.83 |  | |  |
| NBA_1 | - | | 12.79±0.70 | | | 10.96±0.75 | **1.83**** |  | |  |
| NBA | - | | 12.19±0.53 | | | 12.89±0.49 | ***-0.70*** |  | |  |
| NBA_All | - | | 55.08±4.72 | | | 62.49±5.11 | -7.41 |  | |  |
| BALWT_1 | - | | 18.93±1.02 | | | 15.83±1.10 | **3.10**** |  | |  |
| BALWT | - | | 17.83±0.83 | | | 18.89±0.63 | ***-1.06*** |  | |  |
| BALWT_All | - | | 81.83±7.01 | | | 91.46±7.59 | -9.63 |  | |  |
| P_All | - | | 4.50±0.36 | | | 5.01±0.40 | -0.51 |  | |  |
| SNP_8 (rs81312912) | | | | | | | | | | |
| TNB_1 | 12.64±0.40 | | 13.34±0.55 | | | 13.89±1.04 | -1.25 | -0.72 | | +0.47 |
| TNB | 14.04±0.42 | | 14.42±0.41 | | | 13.56±0.68 | 0.48 | 0.24 | | +0.44 |
| TNB_All | 69.00±2.85 | | 66.70±4.32 | | | 75.80±8.82 | -6.80 | -3.40 | | -3.01 |
| NBA_1 | 11.16±0.38 | | 11.14±0.57 | | | 12.00±1.09 | -0.83 | -0.42 | | -0.13 |
| NBA | 12.70±0.35 | | 12.96±0.37 | | | 12.54±0.62 | 0.16 | 0.08 | | +0.22 |
| NBA_All | 62.30±2.51 | | 59.03±3.81 | | | 69.20±7.78 | -6.90 | -3.45 | | -3.99 |
| BALWT_1 | 16.27±0.57 | | 16.17±0.83 | | | 17.00±1.60 | -0.73 | -0.37 | | -0.19 |
| BALWT | 18.59±0.55 | | 18.97±0.55 | | | 18.56±0.92 | 0.03 | 0.02 | | +0.35 |
| BALWT_All | 91.37±3.72 | | 86.57±5.64 | | | 101.40±11.52 | -10.03 | -5.02 | | -5.84 |
| P_All | 5.09±0.19 | | 4.66±0.29 | | | 5.40±0.60 | -0.31 | -0.16 | | ***-0.46*** |
| SNP_9 (rs80962240) | | | | | | | | | | |
| TNB_1 | 13.04±0.34 | | 12.78±0.61 | | | 13.56±0.82 | -0.52 | -0.26 | | -0.38 |
| TNB | 13.94±0.47 | | 14.35±0.42 | | | 14.76±0.56 | ***-0.82*** | ***-0.41*** | | 0.27 |
| TNB_All | 66.08±2.67 | | 73.50±4.77 | | | 68.91±6.19 | -2.83 | -1.42 | | ***6.89*** |
| NBA_1 | 11.11±0.34 | | 10.96±0.63 | | | 12.24±0.84 | -1.14 | -0.57 | | -0.34 |
| NBA | 12.64±0.38 | | 12.94±0.38 | | | 13.22±0.51 | -0.58 | 0.29 | | 0.30 |
| NBA_All | 59.19±2.37 | | 65.87±4.23 | | | 62.09±5.49 | -2.90 | -1.45 | | ***6.14*** |
| BALWT_1 | 16.02±0.50 | | 16.04±0.93 | | | 18.09±1.24 | ***-2.07*** | ***-1.04*** | | -0.32 |
| BALWT | 18.55±0.59 | | 18.96±0.58 | | | 19.20±0.76 | -0.65 | -0.33 | | 0.40 |
| BALWT_All | 86.85±3.51 | | 96.77±6.25 | | | 90.45±8.12 | -3.60 | -1.80 | | ***9.25*** |
| P_All | 4.81±0.18 | | 5.21±0.33 | | | 4.82±0.42 | -0.01 | -0.01 | | ***0.46*** |

** - P ≤ 0.01; * - P ≤ 0.05; bold italics P ≤ 0.15
